# Antioxidant activity and chemical composition of meat from broilers fed diets containing different essential oils

**DOI:** 10.14202/vetworld.2021.1638-1643

**Published:** 2021-06-25

**Authors:** Izabela Lorena Azevedo, Wedson Carlos Lima Nogueira, Anna Christina de Almeida, Lis Lorena Melúcio Guedes, Claudia Regina Vieira, Sergio Henrique Sousa Santos, Carolina Magalhães Caires Carvalho, Francine Souza Alves da Fonseca, Rogério Marcos de Souza, Cintya Neves de Souza

**Affiliations:** Federal University of Minas Gerais, Institute of Agrarian Sciences, Center for Research in Agrarian Sciences, Montes Claros, Minas Gerais, Brazil

**Keywords:** broiler, *Cymbopogon flexuosus*, *Lippia* aff*. rotundifolia*, moisture, storage time

## Abstract

**Background and Aim:**

Broiler meat is susceptible to lipid oxidation due to its high content of unsaturated fatty acids, especially when stored for a long time. Concerning with that problem, we can add natural compounds to the animal feed, as the plant essential oils, which have antioxidant, antimicrobial, and antifungal activities. The objective of this study was to evaluate the antioxidant activity, fatty acid profile, and chemical composition of the meat of broilers fed with diets containing different essential oil, including lemongrass (*Cymbopogon flexuosus*) and pedestrian tea (*Lippia* aff. *rotundifolia*).

**Materials and Methods:**

The experimental design used was a 4×4 factorial scheme (storage time×diets), and each treatment was repeated thrice. The treatments were applied as negative control (without antimicrobial); positive control (ration supplemented with 10 ppm of enramycin and 42 ppm of salinomycin), lemongrass (ration with 120 mg of essential oil of lemongrass for each kilogram of live weight of the animals), and pedestrian tea (ration with 120 mg of essential oil of pedestrian tea for each kilogram of live weight of the animals). Storage was performed after slaughter and evaluated monthly for 4 months.

**Results:**

The inclusion of lemongrass oil and pedestrian tea in the broiler diet did not change the ether extract content of the meat sample obtained from thigh and drumstick. The storage time promotes an increase in the moisture loss of the meat from the 2^nd^ month in all treatments. There was an interaction between the additive and storage time for moisture loss. In the 2^nd^ month of storage, the meat from broilers fed the antibiotic-containing feed had a lower moisture loss than those from broilers in the other treatment groups. The fatty acid profile did not differ statistically between the different treatment groups. The inclusion of additives in the food dropped the lipid oxidation at the 2^nd^ month of storage. From the 3^rd^ month, however, only the essential oils showed antioxidant effect.

**Conclusion:**

Antioxidant activity was positive for treatments that included essential oils. The lemongrass oil and pedestrian tea can be used as antioxidant additives in broiler diets to improve the oxidative stability of thigh-drumstick meat during storage. The results of the study suggest a good prospective for further research with profitability of usage essentials oils examined, for their economical application as feed components in broiler nutrition.

## Introduction

Broiler meat is largely used in most Brazilian meals and is one of the most consumed animal protein sources in the country. The per capita consumption of broiler meat in 2019 was approximately 42 kg/inhabitant/year. This high consumption value is associated with this meat being the cheapest protein and more accessible than fish, and also the poultry meat being considered healthy [1]. Broiler meat is susceptible to lipid oxidation due to its high content of unsaturated fatty acids. Lipid oxidation is accelerated during the handling, processing, storage, and cooking of meat products [2].

The synthetic antioxidants that are most widely used in the food industry as food preservatives are butylhydroxytoluene (BHT), butylhydroxyanisole, tertiary butylhydroxyquinone, and propyl gallate. At present, to meet the increasing consumer demands, there is a tendency to replace synthetic antioxidants with natural compounds that have the same function. Such natural compounds include plant essential oils that have antioxidant, antimicrobial, and antifungal activities [3].

The effect of adding essential oils to animal feed on meat quality is not well understood and raises safety concerns. Because this additive contains complex molecules, it is necessary to understand the synergism and antagonism of its constituents, the optimal concentration of each constituent, the possibility, and mechanism of their absorption by the gastrointestinal tract [4].

Therefore, this study aimed to evaluate the antioxidant activity, fatty acid profile, and chemical composition of thigh and drumstick meat from broiler chickens at 42 days of age fed with ration containing essential oils of lemongrass (*Cymbopogon flexuosus* [CF]) and pedestrian tea (*Lippia* aff. *rotundifolia*).

## Material and Methods

### Ethical approval

The procedures were performed in accordance with ethical standards and approved by the Ethics Committee on Animal Usage, protocol no. 102/2013. The procedures adopted followed those described by Azevedo *et al*. [5].

### Study period and location

The study was conducted from January to May 2015. The research was conducted in Montes Claros, Minas Gerais, Brazil (16°44’06” S, 43°51’43” W).

### Experimental design and bird husbandry

A total of 150 male and female 1-day-old chicks of the Cobb 500 line were housed in cages (60×35×100 cm) with feeders and drinking fountains. The design used was a 4×4 factorial scheme (storage time×diets) in a completely randomized design, with three replicates per treatment. The storage time evaluated ranged from 1 to 4 months. The experimental treatments were as follows:

a) Negative control (NC): No antimicrobials and anticoccidials (NC)
b) Positive control (PC): Feed supplemented with 10 ppm enramycin and 42 ppm salinomycin (PC)
c) Lemongrass: Feed containing 120 mg of CF essential oil for each kilogram of animal body weight (CF)
d) Pedestrian tea: Feed containing 120 mg of *Lippia rotundifolia* (LR) essential oil for each kilogram of animal body weight (LR)

The dose used was defined from the antimicrobial activity presented by the essential oils of LR [6] and lemongrass [7] in preliminary *in vitro* tests.

### Experimental diets

The nutrition plan was divided into three phases: Starter (1-21 days), grower (22-33 days), and finisher (34-42 days), as indicated by Rostagno *et al*. [8]. The diets were formulated to meet nutritional levels recommended by Rostagno *et al*. [8] and offered *ad libitum* throughout the experimental period in a mashed form (Table-1). The metabolizable energy and crude protein levels were, respectively, 3069.92 kcal/kg and 21.25% (starter diet); 3165.93 kcal/kg and 19.84% (grower diet); and 3215.51 kcal/kg and 18.43% (finisher diet).

**Table-1:** Composition of experimental diets.

Ingredients	Initial	Growth	Final
Corn (%)	54.10	57.18	60.26
Soybean meal 46% (%)	37.49	33.75	30.20
Soy oil (%)	4.494	5.163	5.652
Dicalcium phosphate (%)	1.571	1.338	1.200
Calcitic limestone (%)	0.910	0.865	0.778
Common salt (%)	0.483	0.458	0.446
DL-methionine (%)	0.147	0.142	0.132
L-lysine (%)	0.064	0.075	0.102
Choline chloride (%)	0.055	0.050	0.037
Mineral Premix (%) (1)	0.050	0.050	0.050
Vitamin Premix (%) (2)	0.040	0.030	0.020
Inert (%)	0.600	0.900	1.200
Total (%)	100.0	100.0	100.0

**Additives (mg.kg^–1^)**			

Coccidiostat (%) (3)	0.050	0.050	0.050
Antimicrobial (%) (4)	0.012	0.012	0.012
*Cymbopogon flexuosus* essential oil (%) (5)	0.210	0.500	0.633
*Lippia rotundifolia* essential oil (%) (5)	0.210	0.500	0.633

**Calculated content**			

Crude protein (%)	21.25	19.84	18.43
Metabolizable energy (kcal/kg^–1^)	3069.92	3165.93	3215.51
Available phosphorus (%)	0.40	0.35	0.30
Calcium (%)	0.84	0.75	0.66
Sodium (%)	0.21	0.20	0.20
Lysine (%)	1.22	1.13	1.06
Methionine (%)	0.48	0.45	0.43

(1)Each kg of trace mineral premix contains: Cu (min). 15 g.kg^-1^; Fe (min). 100 g.kg^-1^; Mn (min). 140 g.kg^-1^; Zn (min). 100 g.kg^-1^; I (min). 2.400 mg.kg^-1^; Se (min). 400 mg.kg^-1^; inclusion of 500 g/t of feed. (2)Each kg of initial vitamin premix contains: vitamin A (min). 14.000.000.00 IU.kg^-1^; vitamin D3 (min). 4.400.000.00 IU.kg^-1^; vitamin E (min). 22.000.00 IU.kg^-1^; vitamin. K3 (min). 3.200.00 mg.kg^-1^; vitamin B1 (min). 4.000.00 mg.kg^-1^; vitamin B2 (min). 10.000.00 mg.kg^-1^; vitamin B6 (min). 6.000.00 mg.kg^-1^; vitamin B12 (min). 24.000.00 mcg.kg^-1^; niacin (min). 70 g.kg^-1^; pantothenic acid (min). 26 g.kg^-1^; folic acid (min). 1.600.00 g.kg^-1^; inclusion of 500 g/t of feed. Growth Vitamin Premix containing per kg^-1^ of product: vitamin A (min). 12.000.000.00 IU.kg^-1^; vitamin D3 (min). 4.000.000.00 IU.kg^-1^; vitamin E (min). 20.000.00 IU.kg^-1^; vitamin K3 (min). 3.200.00 mg.kg^-1^; vitamin B1 (min). 2.800.00 mg.kg^-1^; vitamin B2 (min). 8.000.00 mg.kg^-1^; vitamin B6 (min). 4.000.00 mg.kg^-1^; vitamin B12 (min). 20.000.00 mcg.kg^-1^; niacin (min). 60 g.kg^-1^; pantothenic acid (min). 22 g.kg^-1^; folic acid (min). 1.200.00 g.kg^-1^. (3)Salinomycin coccidiostat for all phases at 500 g/t of feed. (4)Antimicrobial enramycin for all phases at 125 g/t of feed. (5)Essential oil of *Cymbopogon flexuosus/Lippia rotundifolia* in the corresponding treatments.

The essential oil of lemongrass was purchased from Ferquima Indústria e Comércio LTDA (Vargem Grande Paulista, SP, Brazil) and tea-pedestrian oil was purchased from producers at the Fundação do Vale do Jequitinhonha Cooperativa (Serro, district of São Gonçalo do Rio das Pedras, Minas Gerais, Brazil). Both oils were extracted by steam dragging and packed in 500 mL bottles. The oils were analyzed by a gas chromatograph coupled to a mass spectrometer (CG-EM). Subsequently, the oils were converted into microcapsules by Croma Microencapsulares (São Paulo, SP, Brazil) through coacervation with edible polymers. The microencapsulated essential oils were then mixed to the ration using a manual mixer for small quantities and stored in a freezer. After mixing, the rations were analyzed and the essential oils’ volatile compounds were extracted in the static headspace and analyzed by CG-EM.

### Sample collection and analysis

At 42 days of age, three birds were randomly selected from each experimental plot (±10% average weight), fasted for 8 h, slaughtered by bleeding from the jugular vein, scalded, plucked, eviscerated, and cut. Each bird’s thigh-drumstick was separated for further bromatological analysis, antioxidant activity determination, and fatty acid profiling. The cuts were weighed, cleaned, packed in well-labeled trays, and placed at −18°C in the freezer until the time of analysis (storage conditions 1, 2, 3, and 4 months). Thigh-drumstick was chosen from among the cuts of greatest commercial value in Brazil and their physiological characteristics.

Bromatological analyses of protein and moisture contents were performed according to the method of the Adolfo Lutz Institute [9]. The lipid oxidation of the samples was determined through the Kreis reaction [9] which determines the quality of the meat fat. The methodology for preparing these derivatives was that described in Jham *et al*. [10], with some modifications. The first step was the hydrolysis of the hexane extract triacylglycerides (10.00 mg) with addition of KOH solution in MeOH (1 mL, 0.5 mol/L) and heating at 100°C for 1 h, under reflux. Posteriorly, HCl (400 mL, 36% v/v) in methanol (4: 1, v/v) was added, maintaining the temperature at 100°C, for 1 h. After cooling, the extraction of methyl esters was performed with the addition of distilled H_2_O (2.0 mL) and extracted with CH_2_Cl_2_, dichloromethane, (2×3.0 mL). The organic phase was dried over anhydrous MgSO_4_, filtered, and concentrated on rotary evaporator. The residue obtained, after complete removal of the solvent was redissolved in 1.00 mL of CH_2_Cl_2_ and analyzed by GC–MS. The analysis of fatty acids was carried out according to the method of Azevedo *et al*. [5]

### Statistical analysis

The results obtained from the factorial (diet × storage time) experiments were subjected to analysis of variance using the R Statistical software R Core Team [11], and in the presence of significant effects of the interaction, the means were compared by the Tukey test (p<0.05). The statistical model used was as follows:


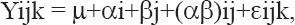


where, Yijk: A set of observations of the dependent variable corresponding to the storage time of the cuts i, addition of essential oils j, and repetition k; µ: A set of observations; αi: Effect of the storage time of the cuts; βj: Effect of the addition of essential oils into the diet; (αβ)ij: Effect of the interaction between storage time of the cuts i and addition of essential oils in the order j; and εijk: Experimental error from the observation of the effect of storage time of the cuts i, adding essential oils in the diet j, and repetition k.

## Results

### Bromatological analysis

The inclusion of lemongrass and pedestrian tea oils in broiler diet did not influence the ether extract content of the cuts compared with that of meat from other treatment groups (Table-2). With respect to storage time, ether extract content of the thigh-drumstick did not change significantly (p=0.150) during the 4 months.

**Table-2:** Analysis of ether extract (EE%) and moisture (MO%) of the broiler thigh/drumstick at 42 days of age.

Additives	EE(%)	MO (%)
NC	4.120±0.80	73.625^b^±0.72
PC	4.579±0.68	74.987^a^±0.56
CF	4.524±0.71	73.770^b^±0.63
LR	4.738±0.88	73.332^b^±0.79
p-value	0.2432	p<0.01
Time (months)		
1	4.575±0.76	75.046^a^±0.38
2	4.755±0.50	73.768^b^±0.33
3	4.624±0.65	73.631^b^±0.25
4	4.066±0.71	73.270^b^±0.30
p-value	0.150	p<0.01
Additives x Time	0.3296	p<0.01
*CV*	16.78	1.72

Mean±standard error standard. Similar superscripts in the same column do not differ significantly (p<0.05). CV: coefficient of variation (NC) Feed negative control without additives; (PC) Feed positive control with antimicrobials and anticoccidials; (CF) Control feed + essential oil of *Cymbopogon flexuosus*; (LR) Control feed + essential oil of *Lippia rotundifolia*

The moisture was higher (p<0.01) in the antibiotic treatment group with than in the NC group, pedestrian tea, and lemongrass groups (Table-2). The storage time contributed to a decrease (p<0.01) in the moisture content of the meat after the 2^nd^ month. There was an interaction between the additive and storage times with regard to moisture content (p<0.01). Table-3 shows that the inclusion of antimicrobials and anticoccidials to the rations improves the moisture of the thigh/drumstick cut during the 2^nd^ month of storage.

**Table-3:** Breakdown of the interaction between diets and storage time (month) for moisture (%) in broilers at 42 days of age.

TRT	1 (month)	2 (month)	3 (month)	4 (month)
NC	75.060^aA^±0.66	73.550^bB^±0.84	74.093^aB^±0.36	73.800^aB^±0.86
PC	75.510^aA^±0.80	75.870^aA^±0.41	73.503^aB^±0.42	74.066^aB^±0.79
CL	74.986^aA^±0.72	73.876^bB^±0.53	73.553^aB^±0.54	73.666^aB^±0.67
CP	74.630^aA^±0.56	72.776^bB^±0.65	73.376^aB^±0.48	72.546^aB^±0.53

Means±standard error followed by lowercase letters differ on the same column and the means ± standard error followed by capital letters differ on the same line (p<0.05). (NC) Feed negative control without additives; (PC) Feed positive control with antimicrobials and anticoccidials; (CF) Control feed + essential oil of *Cymbopogon flexuosus*; (LR) Control feed + essential oil of *Lippia rotundifolia*;

There was no significant difference in meat quality of the different groups in the 1^st^, 3^rd^, and 4^th^ months of storage. However, in the 2^nd^ month, meat from the PC group had a higher moisture content compared with those from the other treatment groups. For all treatment groups, moisture content dropped from the 2^nd^ month of storage.

### Fatty acid profile and lipid oxidation by the Kreis test

The composition of fatty acids did not differ significantly (p>0.05) between meat from the different treatment groups (Table-4). Oleic and linoleic acid contents averaged 38.17% and 25.81%, respectively, in meat from all groups.

**Table-4:** Fatty acid profile of the thigh/drumstick of broilers at 42 days of age.

Fatty acids	NC (%)	PC (%)	CF (%)	LR (%)	p-value	CV
Elaidic (C18:1 trans)	0.80±0.16	0.60±0.29	0.83±0.23	0.63±0.12	0.340	24.83
Hexadecanoic (C16:0)	24.46±0.80	23.20±0.75	24.40±0.62	25.80±0.51	0.198	5.37
Linoleic (C18:2 cis-9 cis-12)	25.26±0.42	25.66±0.54	26.06±0.80	26.26±0.46	0.311	2.51
Oleic (C18:1 cis-9)	38.66±0.45	37.70±0.56	38.23±0.71	38.10±0.64	0.780	2.99
Palmitoleic (16:1n-7)	0.80±0.38	1.30±0.52	0.80±0.20	1.32±0.41	0.280	30.10

(NC) Feed negative control without additives; (PC) Feed positive control with antimicrobials and anticoccidials; (CF) Control feed + essential oil of *Cymbopogon flexuosus*; (LR) Control feed + essential oil of *Lippia rotundifolia*. (CV): coefficient of variation; ^a,b,c^Mean±standard error bearing similar superscripts in the same row does not differ significantly (p<0.05)

Lemongrass oil, pedestrian tea, and antibiotics all exerted antioxidant action on the meat (Table-5). Furthermore, lipid oxidation was absent in meats from the lemongrass and pedestrian tea groups throughout the storage period. The NC and PC groups showed lipid oxidation after the 2^nd^ month of storage.

**Table-5:** Lipid oxidation by the Kreis test of the thigh/drumstick of broilers at 42 days of age.

Time (months)	NC thigh/drumstick	PC thigh/drumstick	CF thigh/drumstick	LR thigh/drumstick
1	n	n	n	n
2	p	n	n	n
3	p	p	n	n
4	p	p	n	n

(NC) Feed negative control without additives; (PC) Feed positive control with antimicrobials and anticoccidials; (CF) Control feed + essential oil of *Cymbopogon flexuosus*; (LR) Control feed + essential oil of *Lippia rotundifolia*; (P) Positive; (N) Negative

## Discussion

The use of essential oils in the feed did not affect the composition of ether extract of the meat. Figueiredo [4] evaluated the bromatological composition of the thigh-drumstick meat of broilers fed with different levels of essential oils of thyme and basil included in the feed. The ether extract was on average 4.51%, similar to the results of this study.

The moisture content was the highest in the 1^st^ month of storage for all treatment groups. These values may be related to water loss to the environment. During the freezing, storage, and thawing processes, the meat loses water through evaporation, sublimation, and exudation, respectively. Evaporative losses depend on freezing conditions, as well as relative humidity and temperature, and the characteristics of the meat, such as the size and area-volume ratio of the pieces, fat cover, and the presence of skin or packaging [12]. From the 2^nd^ month of storage, there was a drop in moisture content in meat from all treatment groups. These results can be explained by the loss of water by evaporation during freezing [12].

The fatty acid composition of the meats from the different treatment groups did not differ statistically. These results are consistent with the findings of Figueiredo [4], who evaluated the fatty acid profile of meat from broiler chickens fed with different levels of essential oils of thyme and basil included in the feed. In that study, the oleic and linoleic acids were on average 37.50% and 20.97%, respectively.

Our results show that the use of phytogenic additives in the feed may prevent lipid oxidation in the meat. Essential oils are rich in phenolic compounds (thymol, carvacrol, and eugenol), which can improve meat quality by reducing lipid oxidation and microbial growth [5,13]. According to Jayasena and Jo [14], these compounds prevent lipid oxidation by acting as free radical scavengers and hydrogen donors.

Luna *et al*. [15] used thymol and carvacrol, which are constituents of essential oils, in the feed of broilers. In that study, the NC group (without antioxidant) was compared with three others: PC (150 mg/kg of BHT), 150 mg/kg thymol, and 150 mg/kg carvacrol. They found lower malonaldehyde levels in the thigh meat after 5 days of refrigeration in the thymol and carvacrol groups. This result indicated that thymol and carvacrol had a positive effect on meat preservation. Fratianni *et al*. [16] also evaluated the use of thyme essential oil in improving the shelf-life of fresh chicken breast meat stored at 4°C. Inhibition of lipid peroxidation and deterioration of sarcoplasmic proteins were observed in the essential oil-treated meat, and the meat quality was maintained even after 2 weeks of storage.

Figueiredo [5] evaluated the antioxidant activity of essential oils in chicken breast, drumstick, and meatballs. The results showed that the addition of essential oils (*Thymus vulgaris* and *Ocimum gratissimum*) to the feed of broilers and processed meat (meatballs) effectively decreased lipid oxidation compared with that in the control group (without antioxidant addition). The antioxidant action observed following the inclusion of phytogenic compounds in the feed of broilers is likely exerted through absorption of the constituents into the body tissues, wherein they inhibit the reactions involved in lipid oxidation [17].

## Conclusion

Essential oils of lemongrass and pedestrian tea can be used as antioxidant additives in broiler feed to improve the oxidative stability of the thigh-drumstick meat during storage. The results of the study suggest a good prospective for further research with profitability of usage essentials oils examined, for their economic application as feed components in broiler nutrition.

## Authors’ Contributions

ILA, WCLN, CRV, RMS, and ACA: Designed the study. WCLN, FSAF, ILA, LLMG, CNS, SMD, and ACA: Collected the data and did the laboratory work. WCLN, CRV, RMS, and ACA: Supervised the study. CMCC, WCLN, FSAF, CRV, and ACA: Analyzed the data and drafted the manuscript. CMCC, WCLN, SHSS, and ACA: Helped in critical review and data representation of the manuscript. All authors read and approved the final manuscript.
